# Bardoxolone methyl analog attenuates proteinuria-induced tubular damage by modulating mitochondrial function

**DOI:** 10.1096/fj.201900217R

**Published:** 2019-08-30

**Authors:** Hajime Nagasu, Yuji Sogawa, Kengo Kidokoro, Seiji Itano, Toshiya Yamamoto, Minoru Satoh, Tamaki Sasaki, Takafumi Suzuki, Masayuki Yamamoto, W. Christian Wigley, Joel W. Proksch, Colin J. Meyer, Naoki Kashihara

**Affiliations:** * Department of Nephrology and Hypertension, Kawasaki Medical School, Kurashiki, Okayama, Japan;; † Department of Medical Biochemistry, Tohoku University Graduate School of Medicine, Sendai, Miyagi, Japan;; ‡ Reata Pharmaceuticals, Irving, Texas, USA

**Keywords:** dh404, Nrf2, reactive oxidative species, inflammation

## Abstract

Multiple clinical studies have shown that bardoxolone methyl, a potent activator of nuclear factor erythroid 2–related factor 2 (Nrf2), is effective in increasing glomerular filtration rate in patients with chronic kidney disease. However, whether an Nrf2 activator can protect tubules from proteinuria-induced tubular damage *via* anti-inflammatory and antioxidative stress mechanisms is unknown. Using an Institute of Cancer Research–derived glomerulonephritis (ICGN) mouse model of nephrosis, we examined the effects of dihydro-CDDO-trifluoroethyl amide (dh404), a rodent-tolerable bardoxolone methyl analog, in protecting the tubulointerstitium; dh404 markedly suppressed tubular epithelial cell damage in the renal interstitium of ICGN mice. The tubular epithelial cells of ICGN mice showed a decrease in the size and number of mitochondria, as well as the breakdown of the crista structure, whereas the number and ultrastructure of mitochondria were maintained by the dh404 treatment. To further determine the effect of dh404 on mitochondrial function, we used human proximal tubular cells *in vitro*. Stimulation with albumin and free fatty acid increased mitochondrial reactive oxygen species (ROS). However, dh404 administration diminished mitochondrial ROS. Our data show that dh404 significantly reduced proteinuria-induced tubular cell mitochondrial damage, suggesting that improved redox balance and mitochondrial function and suppression of inflammation underlie the cytoprotective mechanism of Nrf2 activators, including bardoxolone methyl, in diabetic kidney disease.—Nagasu, H., Sogawa, Y., Kidokoro, K., Itano, S., Yamamoto, T., Satoh, M., Sasaki, T., Suzuki, T., Yamamoto, M., Wigley, W. C., Proksch, J. W., Meyer, C. J., Kashihara, N. Bardoxolone methyl analog attenuates proteinuria-induced tubular damage by modulating mitochondrial function.

Chronic kidney disease (CKD) is a global public health burden, with a high prevalence and high risk of progression to end-stage renal disease, cardiovascular disease, and premature death (1). The mechanism of CKD progression has been extensively studied but has not been fully elucidated.

Proteinuria has been shown to be an independent risk factor for the progression of kidney disease (2). Moreover, a reduction in proteinuria leads to an improvement in the prognosis of kidney function (3). The reabsorption of urinary albumin, lipid, and chemokines by tubular epithelial cells promotes inflammation and fibrosis by inducing the release of cytokines, chemokines, and TGF-β *via* the enhancement of oxidative stress, NF-κB activation, *etc.* in tubular cells. In turn, these factors promote fibrosis of the tubulointerstitium, resulting in end-stage kidney failure (4).

Free fatty acid (FFA) that is bound to albumin has been shown to induce tubular damage. Excess FFAs induce mitochondrial damage and promote the production of mitochondria-derived reactive oxygen species (ROS). The accumulation of excess mitochondrial ROS results in proximal tubular cell damage and induces apoptosis (5). For this reason, mitochondria-protective therapy has a high potential of manifesting efficacy against many kidney diseases involving proteinuria. Using an animal model of spontaneous nephrosis, we also previously demonstrated that selective estrogen receptor modulators manifest nephroprotective effects through the suppression of mitochondrial damage (6).

The Kelch-like ECH-associated protein 1 (Keap1)–nuclear factor erythroid 2–related factor 2 (Nrf2) pathway plays a central role in mediating the response to oxidative stress and host defense through the induction of cytoprotective and antioxidant gene expression upon exposure of cells to oxidative stress and inflammatory stimuli (7). Mitochondrial metabolism plays an important role in cellular immune response, and Nrf2 is a key regulator of mitochondrial biogenesis and the maintenance of mitochondrial function (8–10).

In addition, the activation of Nrf2 has been shown to protect the kidney. In an acute kidney injury model, Nrf2 activation suppressed tubular damage (11, 12), suggesting that Nrf2 activators can suppress the aggravation of acute kidney injury *via* their mitochondria-protective effects. However, in diabetic kidney disease (DKD) and CKD, which are characterized by proteinuria, the question of whether the activation of the Keap1-Nrf2 pathway can improve the prognosis of kidney function *via* direct effects on tubular protection has not been evaluated. The molecular mechanisms of tubular protection through the activation of the Keap1-Nrf2 pathway have also not been elucidated.

We hypothesized that Nrf2 activators may protect tubules from proteinuria-induced tubular damage *via* their anti-inflammatory and antioxidative stress actions and the suppression of mitochondrial damage. To test this hypothesis, we used dihydro-CDDO-trifluoroethyl amide (dh404), a bardoxolone methyl (CDDO-methyl ester) analog that can be used in rodents (13), in Institute of Cancer Research (ICR)–derived glomerulonephritis (ICGN) mice, a mouse model of nephrosis, in order to examine the tubulointerstitium-protective effects of an Nrf2 activator.

## MATERIALS AND METHODS

### Animal models

The experimental protocols (16-124 and 17-085) were approved by the Animal Research Committee of Kawasaki Medical School and were based on the *Guide for the Care and Use of Laboratory Animals* [National Institutes of Health (NIH), Bethesda, MD, USA]. Three-week-old male ICR mice (Clea Japan, Tokyo, Japan) were used as control (*n* = 13). ICGN mice were selected because they show massive proteinuria and progressive renal failure by 20 wk of age. The age at which treatment was started and the treatment period were decided from preliminary experiments. Commencement of the treatment at 6 wk or later appeared unfeasible because of advanced interstitial fibrosis. The Nrf2 activator RTA dh404 (CDDO-dhTFEA) was used at a dose of 10 mg/kg body weight, dissolved in sesame oil, and administered by oral gavage for 3 wk. These mice were divided into the following 3 groups: ICR mice, ICGN mice treated with vehicle (ICGN mice), and ICGN mice treated with RTA dh404 (ICGN+dh404 mice).

In an albumin-loading model, Nrf2-deficient (Nrf2KO) mice having a C57B/6J background were purchased from Riken (Wako, Japan) (14). A Penny Port (Primetech, Tokyo, Japan) was embedded in the back, and the tip of the tube was inserted into the peritoneal cavity. Bovine serum albumin (BSA; MilliporeSigma, Burlington, MA, USA), bound to FFA, was dissolved in saline at a dose of 250 mg/kg body weight and administered through the port at 2 wk.

### Physiologic and biochemical measurement

Physiologic parameters were measured just before the mice were killed at 6 wk of age. Body weight was examined, blood pressure was measured using the tail-cuff method (BP-98A; Softron, Tokyo, Japan), and urine samples after 24-h food withdrawal were collected in metabolic cages. Blood samples were obtained using a 21-gauge needle inserted into the right atrium of mice that were unfed for 24 h. Urinary kidney injury molecule-1 (KIM-1; Abcam, Cambridge, MA, USA) and neutrophil gelatinase-associated lipocalin (NGAL; Abcam) levels were measured using an enzymatic method.

### Histologic analysis and immunohistochemistry

Right kidney tissue was fixed in 4% paraformaldehyde and embedded in paraffin for histologic analysis. Tissue sections (∼2 μm thick) were deparaffinized and stained with periodic acid–Schiff and Masson staining. The interstitial fibrotic area stained blue with Masson staining was quantitatively estimated using a color image analyzer (Keyence, Osaka, Japan). Tissues were also processed for electron microscopy (Jem-1400; Jeol, Tokyo, Japan) to assess ultrastructural alterations in the tubular mitochondria (6).

Deparaffinized kidney sections (4 μm thick) were heated in a microwave at 500 W, 15 min for antigen retrieval and then incubated overnight with antibody against KIM-1 (R&D Systems, Minneapolis, MN, USA), F4/80 (Bio-Rad, Hercules, CA, USA), collagen IV (Abcam), and podocin (Abcam). The primary antibody was detected using the Histofine Simple Stain Max Peroxidase Kit (Nichirei, Tokyo, Japan) and 3,3′-diaminobenzidine (MilliporeSigma). The percentages of Masson blue–positive area and positive staining for KIM-1, F4/80, and collagen IV were quantified using a Color Image Analyzer (Keyence).

### Real-time quantitative RT-PCR

Total RNA extraction and real-time quantitative RT-PCR were performed, as previously described in Satoh *et al.* (15) Briefly, total RNA was isolated from the kidneys using Trizol (Thermo Fisher Scientific, Waltham, MA, USA), followed by digestion with DNase (MilliporeSigma). cDNA was synthesized from total RNA (1 μg) using Moloney murine leukemia virus reverse transcriptase (Thermo Fisher Scientific) with oligo(dT)_12–18_ as a primer (Thermo Fisher Scientific). Reverse transcription was performed for 50 min at 37°C according to the manufacturer’s protocol (Thermo Fisher Scientific). The primers and probes for TaqMan analysis were designed using sequence information from GenBank (NIH; *https://www.ncbi.nlm.nih.gov/genbank/*) (16) and Primer3 (*http://frodo.wi.mit.edu/primer3*). The primer and probe sequences are listed in Supplemental Table S1. Takara Premix Ex Taq (Takara Bio, Kusatsu, Japan), with a final reaction volume of 20 µl, was used for the TaqMan probe-based RT-PCR reaction, which was performed on an Applied Biosystems 7500 Fast Real-Time PCR System (Thermo Fisher Scientific). Plasmid cDNA of each gene was used to prepare absolute standards. The mRNA expression levels of each gene were normalized to those of the housekeeping 18S rRNA gene.

### Mitochondrial enzyme activity

To identify abnormal mitochondrial enzyme activity, frozen sections (10 µm thick) of the whole kidney were histochemically stained for cytochrome *c* oxidase (COX) and succinate dehydrogenase (SDH) enzyme activities using standard protocols, as previously described in ref. 6.

### TUNEL and caspase-3 staining

Cell death was detected from frozen sections with TUNEL staining (*In Situ* Apoptosis Detection Kit; Takara Bio). Caspase-3 (Abcam) was detected with immunofluorescence staining. Fluorescence was detected using a laser-scanning confocal microscope (Keyence).

### Cell culture

Primary human proximal tubule epithelial cells (hPTECs; Lonza, Basel, Switzerland) were cultured in a renal epithelial basal medium (Lonza) supplemented with 5% fetal bovine serum. After starvation, subconfluent cells (0.5% fetal bovine serum for 24 h) were stimulated with 0.5% BSA (MilliporeSigma) binding 450 μM palmitic acid (BSA+FFA) for 6 h. RTA dh404 (200 or 400 nM) was added to the culture medium 30 min before stimulation with BSA+FFA.

### Luciferase assay

Nrf2 activity was quantified using an antioxidant response element (ARE) reporter HepG2 cell line (BPS Bioscience, San Diego, CA, USA). Cells were cultured in minimal essential medium supplemented with 10% fetal bovine serum. Luciferase activity was measured using the One-Step Luciferase Assay System (BPS Bioscience), and fluorescence intensity was measured using FluoStar Optima (BMG Labtech, Ortenberg, Germany). Cells were transfected with Nrf2 small interfering RNA (siRNA) (Santa Cruz Biotechnology, Dallas, TX, USA) or control siRNA for 24 h by using Lipofectamine 2000 (Thermo Fisher Scientific) according to the manufacturer’s protocol.

### Mitochondrial superoxide detection

After stimulation, mitochondrial superoxide production was visualized using MitoSox Red (Thermo Fisher Scientific). Cells were stained with MitoSox Red for 10 min at 37°C.

### Mitochondrial membrane potential

Mitochondrial membrane potential in live cells was detected using a tetramethylrhodamine ethyl ester (TMRE)–Mitochondrial Membrane Potential Assay Kit (Abcam). Cells were stained for 10 min at 37°C, and mitochondrial membrane potentials were measured with a flow cytometer (BD FACSCanto II; BD Biosciences, San Jose, CA, USA).

### Statistical analyses

Data are expressed as means ± sem. Statistical analyses were performed using Prism 7 software (GraphPad Software, La Jolla, CA, USA). Statistical significance was evaluated using a 1-way ANOVA with Tukey-Kramer *post hoc* tests for comparisons among multiple groups. Values of *P* < 0.05 were considered statistically significant.

## RESULTS

**Figure 1:**
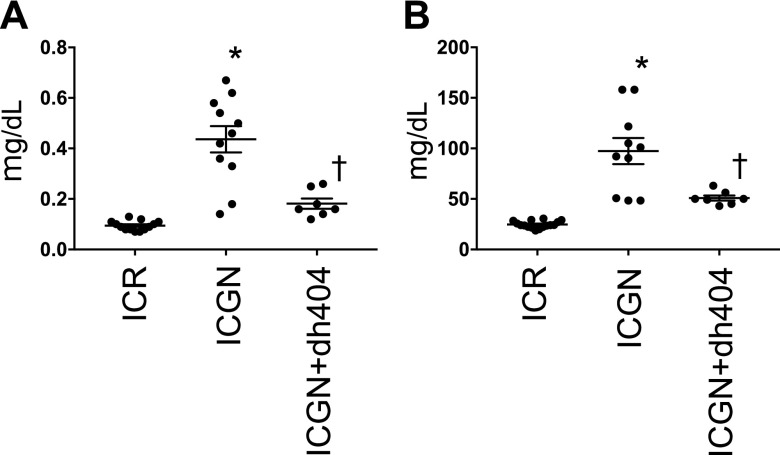
Kidney function in the ICR, ICGN, and ICGN+dh404 groups. *A*) Serum creatinine levels (mg/dl). *B*) Blood urea nitrogen levels (mg/dl). Data are expressed as means ± sem. *P < 0.05 *vs.* ICR, ^†^*P* < 0.05 *vs.* ICGN.

ICGN mice are a model of kidney disease involving the mutation of the tensin 2 gene. These mice develop glomerular damage, particularly podocyte damage, and are characterized by severe proteinuria. Compared with ICR mice, ICGN mice show elevated serum creatinine and urea nitrogen, as well as decreased kidney function. The administration of dh404 significantly lowered serum creatinine and urea nitrogen (**Fig. 1*A, B***).

**Figure 2:**
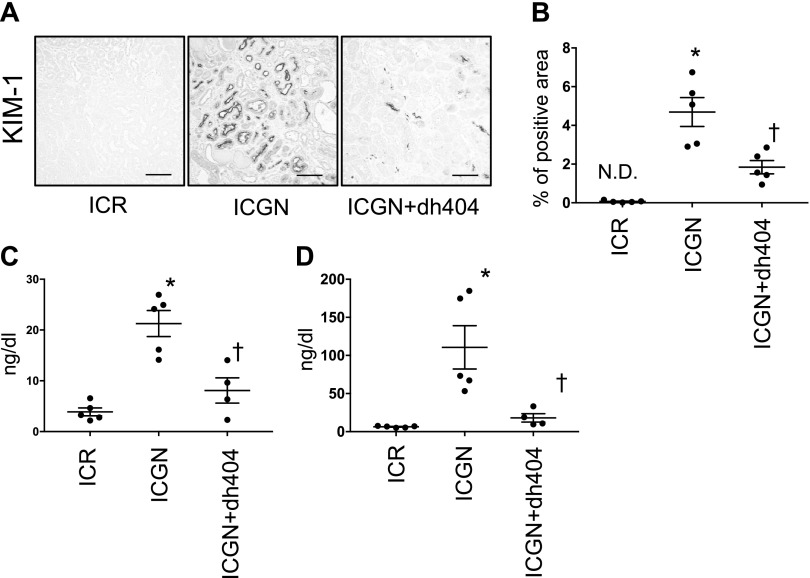
Pathologic changes in tubular epithelial cells. *A*) Immunohistochemical staining for KIM-1 in tubular epithelial cells. Scale bars, 100 µm. *B*) Percentage of KIM-1–positive area. *C*, *D*) Urinary sample from 3 wk after RTA dh404 treatment: KIM-1 (ng/dl) excretion (*C*) and NGAL (ng/dl) excretion (*D*). N.D., not detectable. Data are expressed as means ± sem. *P < 0.05 *vs.* ICR, ^†^*P* < 0.05 *vs.* ICGN.

**Figure 3:**
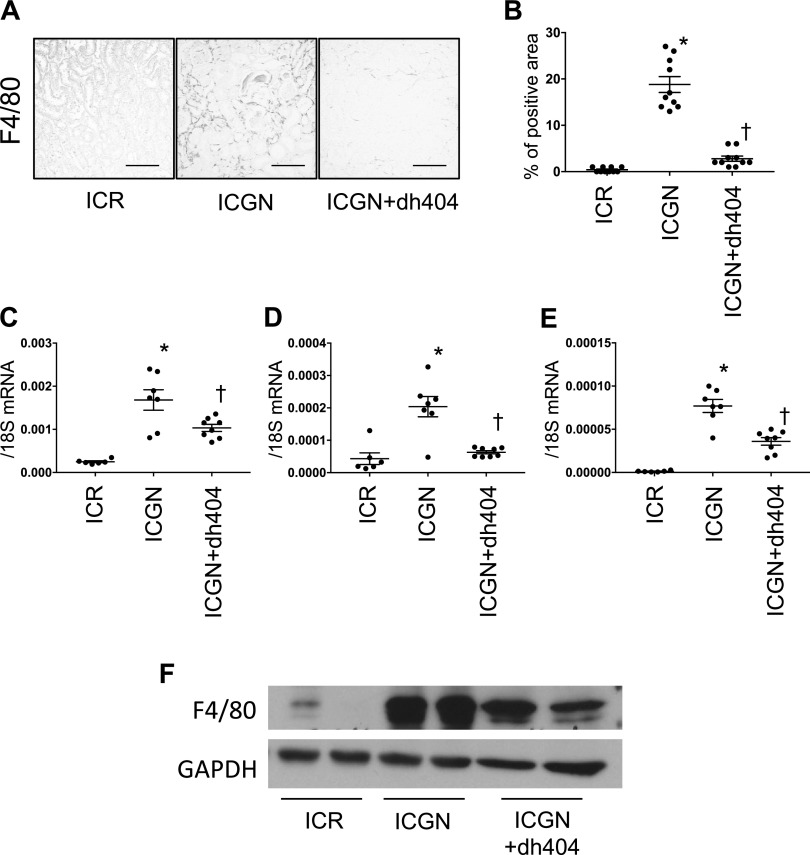
Proinflammatory changes in the renal tubulointerstitium. *A*) Immunohistochemical staining for F4/80 showing infiltrated macrophages in the tubulointerstitium. Scale bar, 100 µm. *B*) Percentage of F4/80-positive area. *C*–*E*) mRNA expression of proinflammatory genes: F4/80 (*C*), IL-6 (*D*), and monocyte chemoattractant protein 1 (*E*). *F*) Western blotting for F4/80 shown as representative data. GAPDH, glyceraldehyde 3-phosphate dehydrogenase. Data are expressed as means ± sem. *P < 0.05 *vs.* ICR, ^†^*P* < 0.05 *vs.* ICGN.

**Figure 4:**
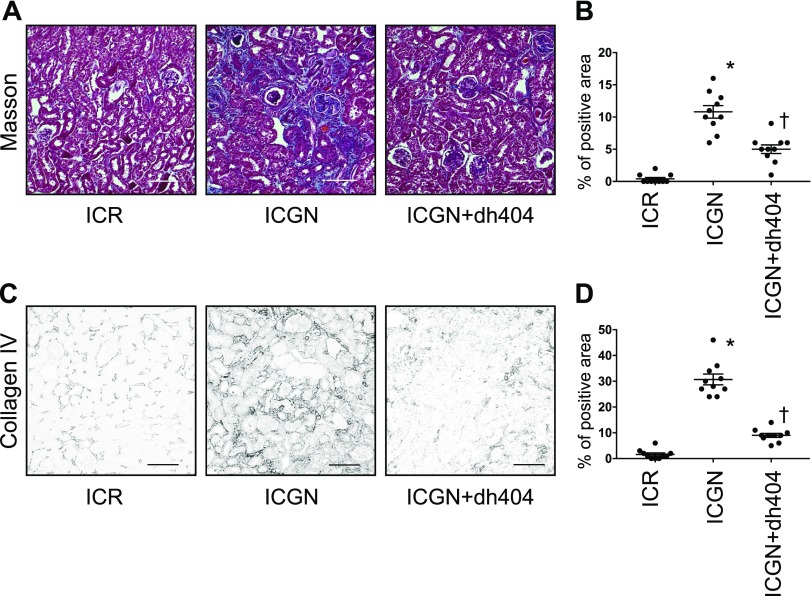
Fibrotic changes in the renal tubulointerstitium. *A*) Masson staining showing the morphology of the renal tubulointerstitium. Scale bars, 100 µm. *B*) Percentage of aniline blue–positive area. *C*) Immunohistochemical staining for collagen IV in the renal tubulointerstitium. Scale bars, 100 µm. *D*) Percentage of collagen IV–positive area. Data are expressed as means ± sem. *P < 0.05 *vs.* ICR, ^†^*P* < 0.05 *vs.* ICGN.

Next, we examined tubular damage by analyzing the changes in KIM-1 expression in tubules. In ICGN mice, the percentage of KIM-1–positive area increased significantly compared with that in ICR mice, whereas in ICGN+dh404 mice, tubular damage decreased significantly (**Fig. 2*A, B***). At the same time, urinary KIM-1 and urinary NGAL concentrations were significantly elevated in ICGN mice compared with ICR mice, but they showed a significant reduction upon the administration of dh404 (Fig. 2*C, D*). The activation of Nrf2 is known to have an inhibitory effect on inflammation. Therefore, we evaluated macrophage infiltration by F4/80 staining and western blotting, and analyzed mRNA levels with quantitative PCR. In ICGN mice, macrophage infiltration increased in the renal interstitium compared with ICR mice, but it was significantly suppressed in ICGN+dh404 mice (**Fig. 3*A–C*, *F***). The expression of IL-6 and monocyte chemoattractant protein 1, which are important for macrophage infiltration, was increased in ICGN mice compared with ICR mice, but it was significantly suppressed in ICGN+dh404 mice (Fig. 3*D, E*). Fibrosis, a common pathway in the progression of kidney diseases, was assessed with Masson’s trichrome (Masson) staining and collagen IV immunostaining. In ICGN mice, fibrosis in the interstitium progressed with the disease (**Fig. 4*A–D***). Furthermore, dh404 treatment suppressed interstitial fibrosis in ICGN mice significantly (Fig. 4*A–D*). Taken together, these results demonstrate that the administration of dh404 in ICGN mice suppresses tubular epithelial cell damage and that this process is accompanied by a suppression of chronic inflammation and progression of fibrosis in the renal interstitium.

**Figure 5:**
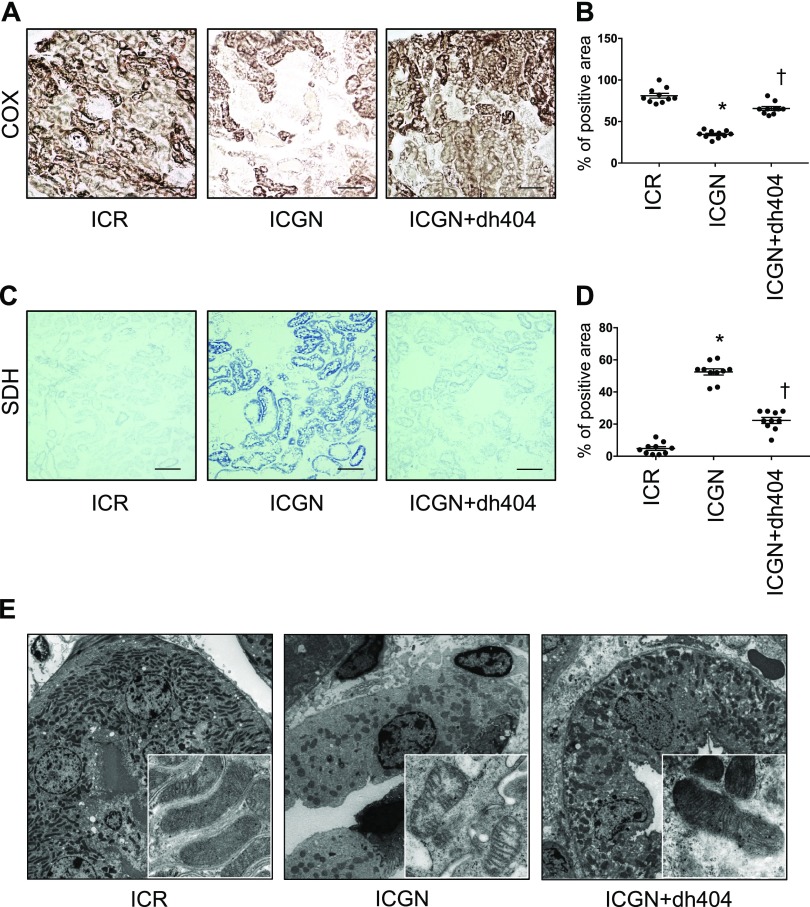
Comparison of mitochondrial function and morphology *in vivo*. *A*) COX activity staining. Scale bars, 100 μm. *B*) Percentage of COX-positive area. *C*) SDH activity staining. Scale bars, 100 μm. *D*) Percentage of SDH-positive area. *E*) Transmission electron microscopy for the evaluation of mitochondrial morphology in tubular epithelial cells. Data are expressed as means ± sem. *P < 0.05 *vs.* ICR, ^†^*P* < 0.05 *vs.* ICGN.

**Figure 6:**
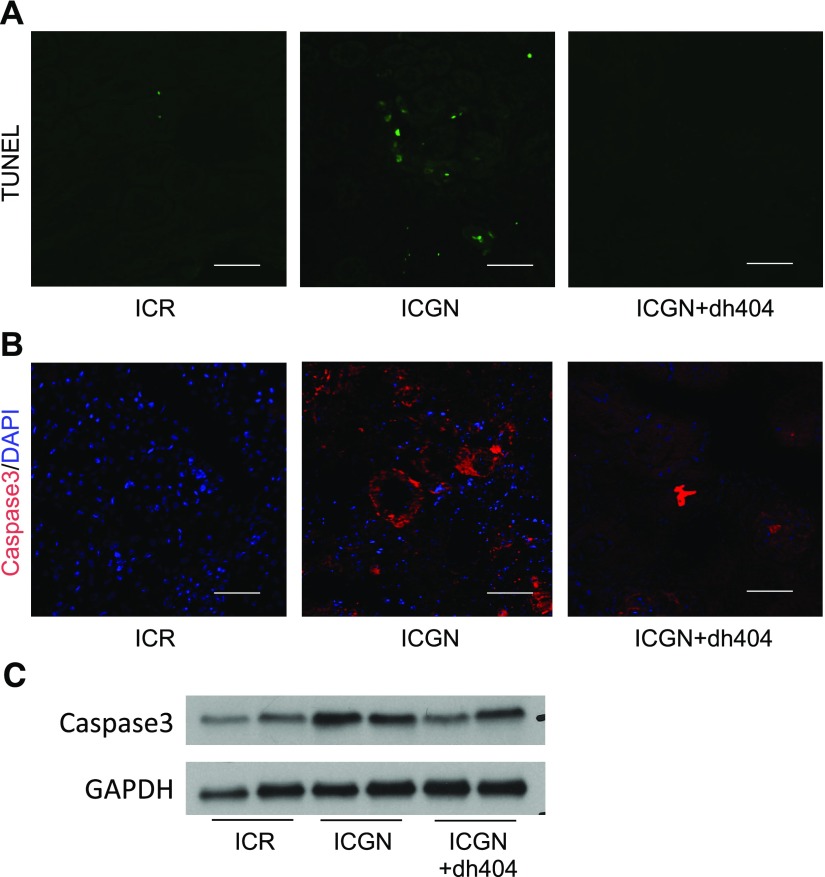
Apoptotic changes in tubular epithelial cells. *A*, *B*) TUNEL staining (*A*) and caspase-3 immunofluorescence staining (*B*) for apoptotic cells. Scale bar, 100 μm. *C*) Western blotting for caspase-3 shown as representative data. GAPDH, glyceraldehyde 3-phosphate dehydrogenase.

We have previously reported the importance of tubular mitochondrial damage in a number of kidney disease models (15, 17). Therefore, changes in respiratory chain function, as an indicator of mitochondrial damage, were assessed with the histochemical measurement of COX (complex IV) and SDH (complex II) activities. COX is a large transmembrane protein that acts as a terminal acceptor in the electron transport chain. It is one of the key enzymes modulating mitochondrial functions. On the other hand, SDH is entirely encoded by nuclear DNA, and its activity is typically not affected by impaired mitochondrial DNA, although an increase might indicate mitochondrial biogenesis. ICGN mice showed a decrease in the number of COX-positive cells and an increase in the number of SDH-positive cells (**Fig. 5*A–D***). Treatment with dh404 improved these mitochondrial electron transport system abnormalities (Fig. 5*A–D*). Changes in mitochondrial ultrastructure in tubular epithelial cells were also analyzed using transmission electron microscopy. The tubular epithelial cells of ICGN mice showed a decrease in the size and number of mitochondria, as well as the breakdown of the crista structure (Fig. 5*E*). By contrast, in ICGN+dh404 mice, the number and ultrastructure of mitochondria were maintained (Fig. 5*E*). In general, mitochondrial damage is known to be related to the apoptosis of tubular cells. Therefore, apoptosis was evaluated with TUNEL staining and caspase-3 staining. In ICGN mice, TUNEL stain–positive tubular epithelial cells and caspase-3–positive cells increased compared with those in ICR mice, showing the induction of apoptosis (**Fig. 6*A, B***). The administration of dh404, by contrast, suppressed apoptosis-related tubular epithelial cell death (Fig. 6*A, B*), consistent with an Nrf2-mediated protection of mitochondrial health.

**Figure 7:**
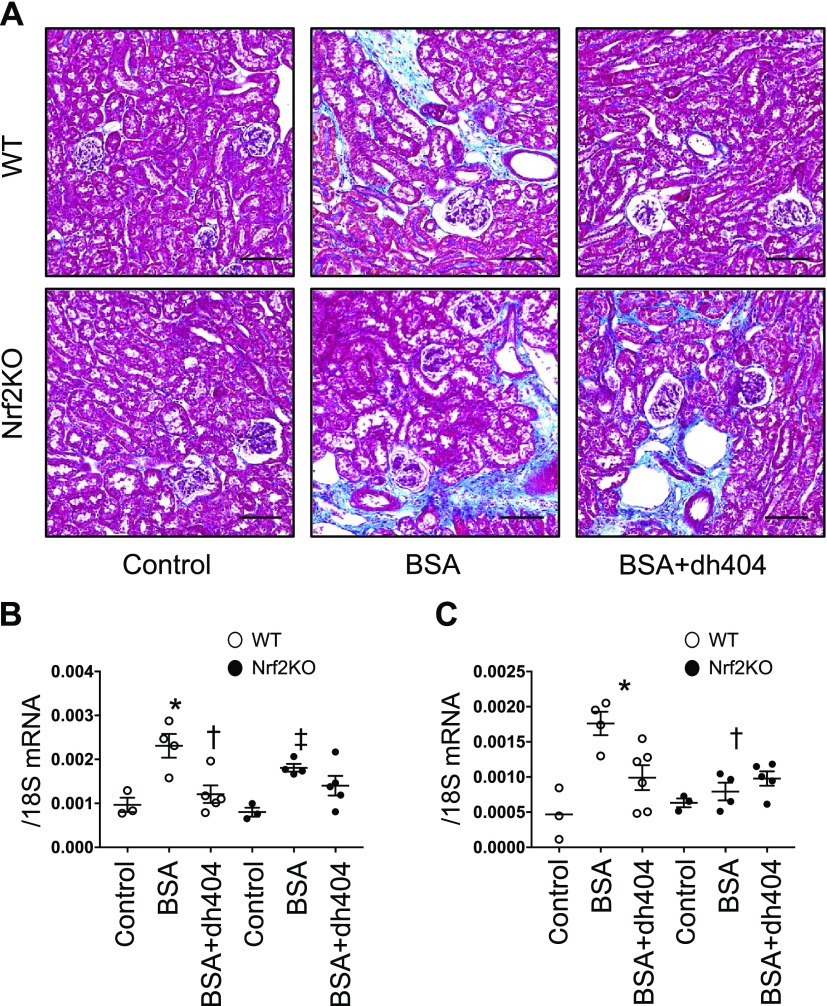
Fibrotic changes in the albumin-loading model. *A*) Masson staining showing renal tubulointerstitial morphology. Scale bars, 100 μm. *B*, *C*) mRNA expression of profibrotic genes: α-smooth muscle actin (*B*) and connective tissue growth factor (*C*). Intraperitoneal injection of BSA injection. Data are expressed as means ± sem. *P < 0.05 *vs.* WT, ^†^*P* < 0.05 *vs.* BSA-WT, ^‡^*P* < 0.05 *vs.* Nrf2KO.

To confirm whether the renoprotective effect of dh404 is mediated by Nrf2 activation, we also studied its effect in Nrf2KO mice. Tubular damage induced by albumin was evaluated using an albumin-loading model (18). The intraperitoneal administration of BSA to wild-type (WT) and Nrf2KO mice promoted fibrosis of the tubulointerstitium. Interestingly, although the administration of dh404 markedly improved fibrosis in WT mice, it did not change fibrosis in Nrf2KO mice, demonstrating that the effects of dh404 are mediated by Nrf2 activation (**Fig. 7*A***). The expression of fibrosis-related genes α-smooth muscle actin and connective tissue growth factor was also assessed. Although albumin loading increased the expression of these genes in both WT and Nrf2KO mice compared with control mice, the administration of dh404 showed an inhibitory effect on gene expression only in WT mice (Fig. 7*B, C*). These results provide *in vivo* evidence that dh404 exerts a renoprotective effect *via* Nrf2 activation.

Furthermore, Nrf2 activation by dh404 was examined using HepG2 ARE luciferase reporter cells. Treatment with dh404 promoted nuclear transfer of Nrf2 in a dose-dependent manner. ARE-dependent luciferase activity was reduced after treatment with Nrf2 siRNA (Supplemental Fig. S1*A, B*). Based on these findings, the concentrations of dh404 administration in the following experiments were set at 200 and 400 nM.

**Figure 8:**
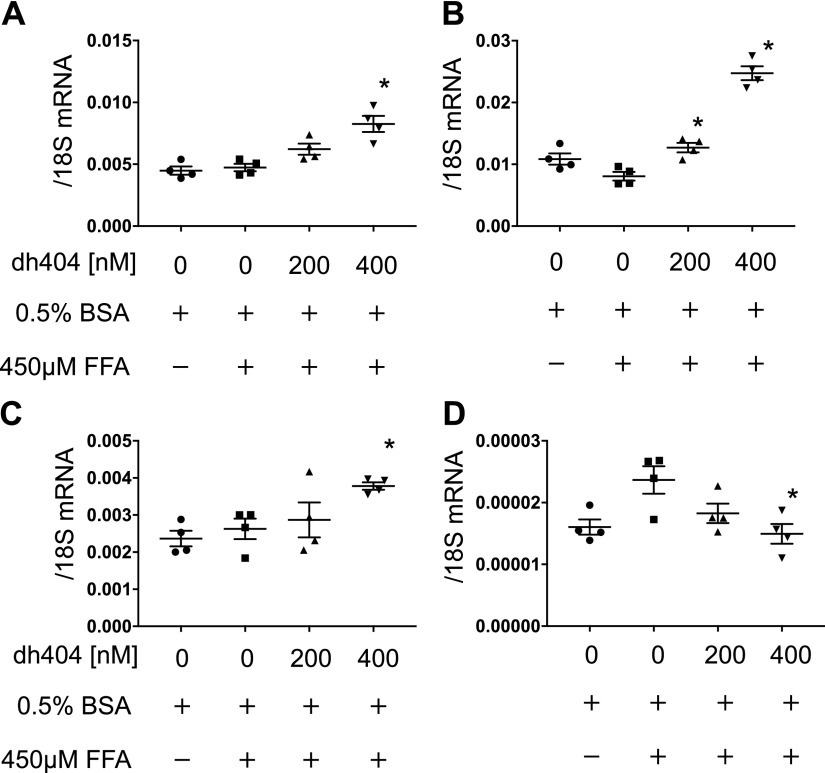
Nrf2-related and anti-inflammatory gene expression *in vitro*. Human proximal tubular cells were used for the *in vitro* assay. mRNA expression of Nrf2-related and anti-inflammatory genes: NQO-1 (*A*), HO-1 (*B*), GCLM (*C*), and IL-1β (*D*). Data are expressed as means ± sem. *P < 0.05 *vs.* control.

The treatment of hPTECs with dh404 increased the expression of a group of Nrf2-targeted antioxidant genes, including NAD(P)H quinone oxidoreductase 1 (NQO-1), glutamate-cysteine ligase modifier subunit (GCLM), and heme oxygenase-1 (HO-1) (**Fig. 8*A–C***). The expression of IL-1β gene was increased in the presence of BSA+FFA, whereas it was suppressed by the administration of dh404 (Fig. 8*D*).

**Figure 9:**
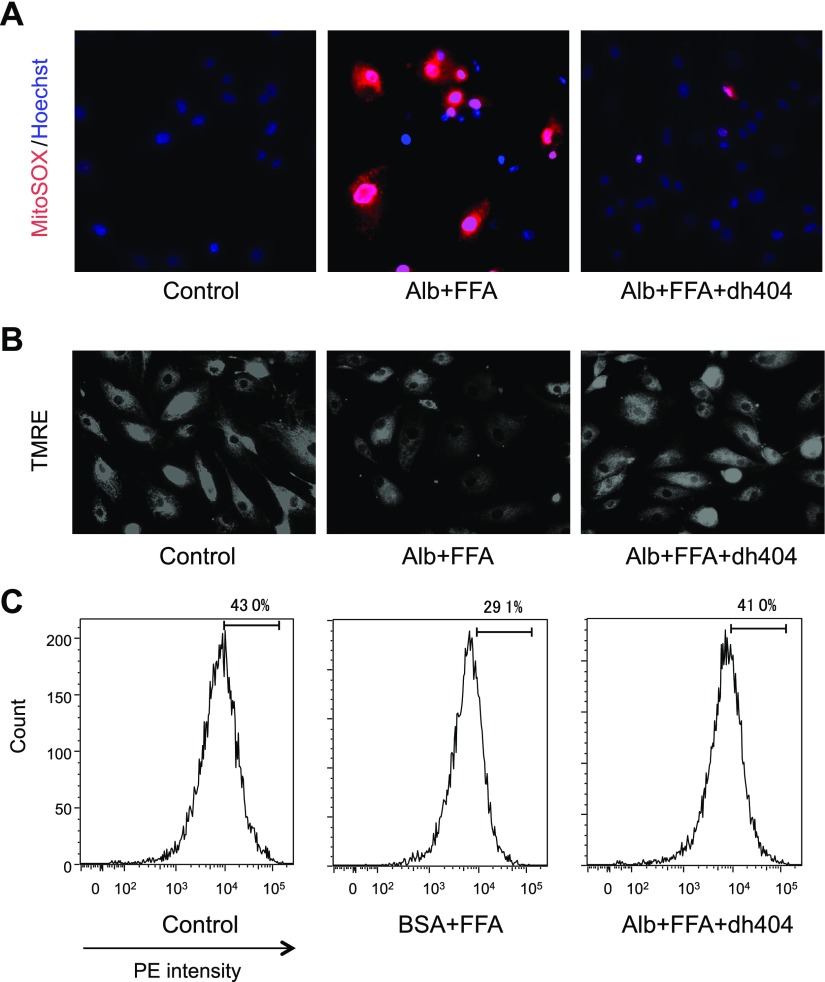
Comparison of mitochondrial ROS and function *in vitro*. Human proximal tubular cells were used for the *in vitro* assay. *A*) MitoSox staining for the evaluation of mitochondrial ROS. The red color shows mitochondrial ROS, and the blue color shows staining of the nucleus by Hoechst stain. *B*) TMRE staining for the evaluation of mitochondrial function. *C*) TMRE staining for the evaluation of mitochondrial membrane potential with flow cytometry. Alb, albumin; PE, phycoerythrin.

Elevated mitochondrial ROS is associated with cellular inflammation and CKD. We next performed MitoSox staining to assess the effect of dh404 on mitochondrial ROS. Stimulation of hPTECs with BSA+FFA produced strong staining with MitoSox, demonstrating increased mitochondrial ROS (**Fig. 9*A***). However, the administration of dh404 diminished MitoSox staining, an observation that was further confirmed with flow cytometry. Because mitochondrial membrane potential is decreased in damaged mitochondria, the depolarization of mitochondrial membrane potential was assessed with TMRE staining, which showed that stimulation by BSA+FFA caused a decrease in mitochondrial membrane potential, which was largely preserved by the administration of dh404 (Fig. 9*B, C*).

## DISCUSSION

Multiple clinical studies have shown that bardoxolone methyl, a potent Nrf2 activator, is effective in increasing glomerular filtration rate in patients with DKD and CKD (19).

Although a phase III study in patients with CKD with concomitant type 2 diabetes was discontinued because of an increase in the risk of heart failure within the first 4 wk of treatment, a *post hoc* analysis identified that the clinical phenotype was acute fluid overload without evidence of cardiotoxicity that occurred in an identifiable subset of patients (20). Clinical development of bardoxolone methyl is currently underway in patients with Alport syndrome (Cardinal, NCT03019185), as well as other rare forms of CKD (Phoenix, NCT03366337), and in patients with CKD with concomitant type 2 diabetes (Ayame, NCT03550443).

Because bardoxolone methyl exhibits tolerability issues in rodents (13), our study used dh404, a closely related analog, and tested the hypothesis that in proteinuria-induced tubular damage, an Nrf2 activator protects tubules from damage *via* anti-inflammatory and antioxidative stress effects and the suppression of mitochondrial damage. In the presence of severe proteinuria, enhanced protein reabsorption in the tubules *via* megalin and cubilin activates NF-κB and becomes a burden to the cells, ultimately causing mitochondrial damage (5, 21). In the present study, dh404 markedly suppressed tubular epithelial cell damage, mitochondrial damage, and inflammation and fibrosis of the interstitium; dh404 also improved kidney function in ICGN mice. However, periodic acid–Schiff staining showed no significant differences in glomerular damage between the ICGN and ICGN+dh404 groups (Supplemental Fig. S2*A, C, D*) or in glomerular epithelial cell damage, as assessed by podocin staining (Supplemental Fig. S2*B*). It is not surprising that in a genetic model of disease, which results in glomerular damage, activation of the pathway (Nrf2) may not impact the underlying pathogenic lesion or the resulting glomerular pathology. Rather, by preserving mitochondrial function, suppressing inflammatory signaling, and reducing oxidative stress, dh404 is protective of the renal tubular interstitium, decreases fibrosis, and preserves kidney function. In *in vitro* experiments, dh404 reduced the excess production of mitochondrial ROS in tubular cells and also preserved mitochondrial membrane potential. Based on these results, dh404 could improve both abnormal tubular mitochondrial function and the long-term prognosis of kidney function.

ICGN mice, the strain used in this study, developed severe proteinuria and inflammation and fibrosis of the renal interstitium (22). dh404 possibly inhibited the transition to fibrosis *via* inflammation suppression. Nrf2 activation has been reported to suppress inflammation in various diseases. For example, there have been reports that Nrf2 activation suppresses macrophage infiltration and is involved in inflammation in emphysema (23, 24) and in a model of pancreatitis (25); dh404 has also been reported to suppress fibrosis and inflammation of the interstitium in a subnephrectomy model, a model of progressive kidney damage (26). The nuclear translocation of Nrf2 has been reported to directly suppress the expression of inflammatory cytokine genes such as IL-6 by binding their promoters and inhibiting their transcription (27). In the present analysis, IL-1β gene expression was also suppressed in hPTECs, suggesting the possibility that dh404 may suppresses the expression of inflammatory cytokine genes in tubular cells by a similar direct mechanism.

Nrf2 binds to the ARE on DNA and promotes the expression of antioxidant genes, such as NQO-1, HO-1, and GCLM. Through the action of such antioxidant enzymes, Nrf2 activation exerts a cytoprotective effect (28–30). Increased ROS induce inflammation and act as an exacerbating factor for organ damage. We have also demonstrated that oxidative stress promotes the progression of kidney diseases (15, 31). In ICGN mice, albumin, particularly FFA-bound albumin, increases tubular mitochondrial ROS (6). Nrf2 activators are effective in various kidney diseases involving oxidative stress (32). In a DKD model, an Nrf2 activator improved not only glomerular lesions but also interstitial damage *via* the elevated expression of antioxidant genes (33). Enhanced antioxidant capacity likely contributes to the renoprotective mechanism of dh404 observed in the present study.

The Keap1-Nrf2 pathway also plays an important role in maintaining mitochondrial function. Nrf2 activation enhances the respiratory function of mitochondria, and Nrf2 deficiency worsens it (34). The maintenance of mitochondrial function in tubular cells is thought to be one of the renoprotective mechanisms of dh404. In our study, tubular cell injury evaluated using KIM-1 expression and other indices was ameliorated by the administration of dh404, along with an improvement of tubular epithelial cell mitochondrial disorders. Therefore, the maintenance of mitochondrial function can be considered a mechanism of renal protection.

In many kidney diseases, tubular epithelial cell damage, chronic inflammation of the tubulointerstitium, and mitochondrial damage are major factors in the progression of kidney damage (17, 35, 36) and are important therapeutic targets.

dh404, a bardoxolone methyl analog, significantly reduced tubular mitochondrial damage caused by proteinuria and improved kidney function. In the future, Nrf2-activating drugs may be promising drugs for the treatment of CKD.

## Supplementary Material

This article includes supplemental data. Please visit *http://www.fasebj.org* to obtain this information.

## Abbreviations

ARE: antioxidant response element
BSA: bovine serum albumin
CKD: chronic kidney disease
COX: cytochrome c oxidase
dh404: dihydro-CDDO-trifluoroethyl amide
DKD: diabetic kidney disease
FFA: free fatty acid
GCLM: glutamate-cysteine ligase modifier subunit
HO-1: heme oxygenase-1
hPTEC: human proximal tubule epithelial cell
ICGN: ICR-derived glomerulonephritis
ICGN+dh404: ICGN mice treated with RTA dh404
ICR: Institute of Cancer Research
Keap1: Kelch-like ECH-associated protein 1
KIM-1: kidney injury molecule-1
NGAL: neutrophil gelatinase-associated lipocalin
NQO-1: NAD(P)H quinone oxidoreductase 1
Nrf2: nuclear factor erythroid 2–related factor 2
Nrf2KO: Nrf2 deficient
ROS: reactive oxygen species
SDH: succinate dehydrogenase
siRNA: small interfering RNA
TMRE: tetramethylrhodamine ethyl ester
WT: wild type
