# Supplementary figures and images for: Bardoxolone methyl analog attenuates proteinuria-induced tubular damage by modulating mitochondrial function
Source: FASEB J. 2019 Aug 30;33(11):12253–63. doi: 10.1096/fj.201900217R (PMC6902727; doi:10.1096/fj.201900217R)

Supplementary Figure S1

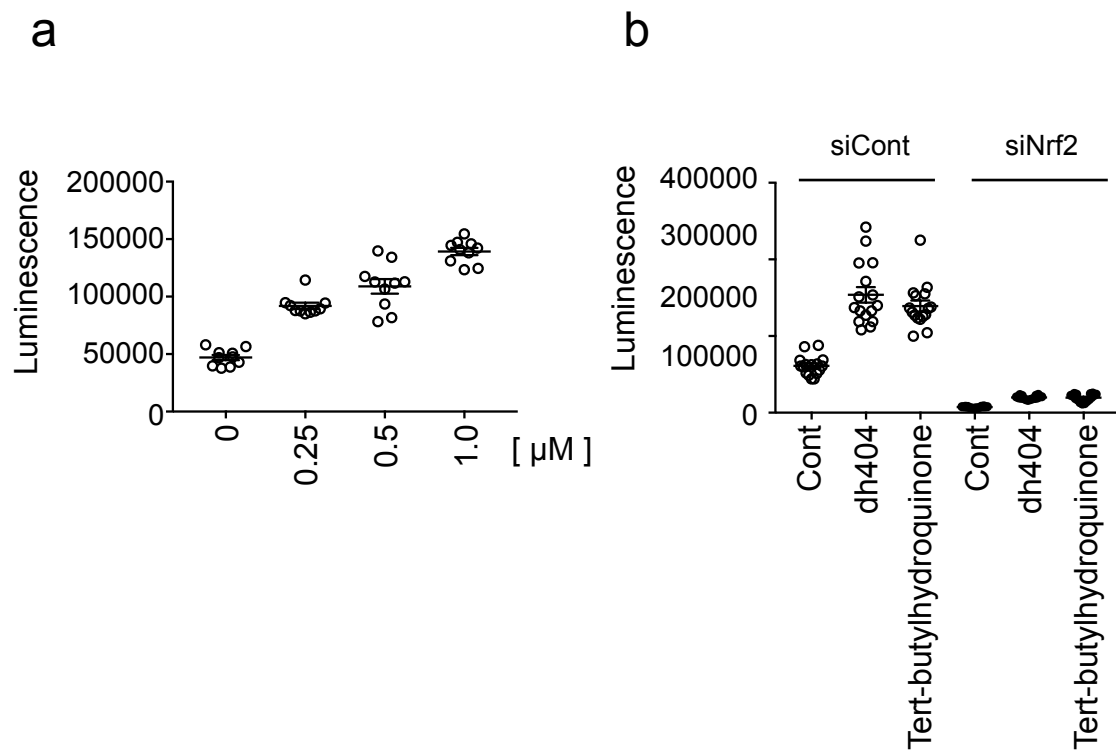

Supplement: Supplementary file 1 [file fj.201900217R.sf1.pdf]

## Supplementary Figure S2

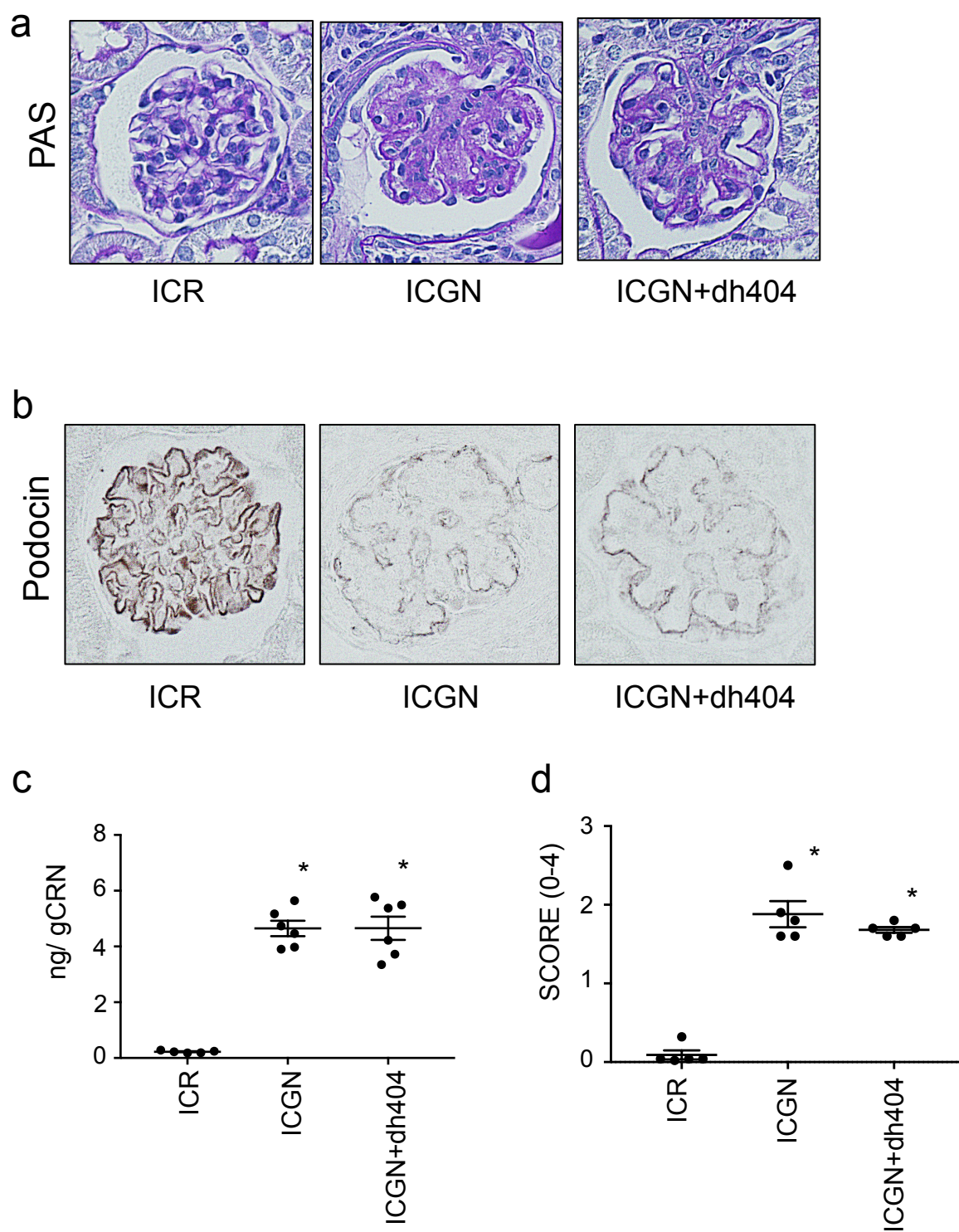

Supplement: Supplementary file 2 [file fj.201900217R.sf2.pdf]
