# Supplementary material for: Bardoxolone methyl analog attenuates proteinuria-induced tubular damage by modulating mitochondrial function
Source: FASEB J. 2019 Aug 30;33(11):12253–63. doi: 10.1096/fj.201900217R (PMC6902727; doi:10.1096/fj.201900217R)
Supplement: Supplementary file 3 [file fj.201900217R.sd1.docx]

Supplementary information is available at FASEB journal’s website.

Supplementary Table 1

**Supplementary Figure S1 RTAdh404 gradually activated by the Nrf2-dependent pathway.** Luminescence intensity was measured using luciferase enzyme assay. (a) ARE reporter cell lines were treated with RTAdh404 (0, 0.25, 0.5, and 1.0 μM). (b) Tert-butylhydroquinone was used as a positive control. ARE, antioxidant response element; Cont, control treated with 0.1% DMSO; DMSO, dimethyl sulfoxide; siCont, transfected with control-siRNA; siNrf2, transfected with Nrf2-siRNA.

**Supplementary Figure S2 Pathological changes in the glomeruli.** (a) PAS staining showing glomerular morphology. (b) Immunohistochemical staining for podocin showing podocyte. (c) Urinary albumin excretion (ng/g CRN). (d) Glomerular injury score. Data are expressed as mean±SEM. *P < 0.05 vs. ICR. CRN, creatinine; ICGN, ICR-derived glomerulonephritis; ICGN+dh404, ICGN mice treated with RTAdh404; ICR, Institute of Cancer Research; PAS, periodic acid-Schiff staining; SEM, standard error of the mean.

Supplement table

| Species | Gene | Accession number | Primer and TaqMan probe sequences (5'‑3') |
| --- | --- | --- | --- |
| mouse | F4/80 | NM_010130 | Forward primer: CCTGGCTTTGCATCTAGCA |
|  |  |  | Reverse primer: AGGAGCCTGGTACATTGGTG |
|  |  |  | TaqMan probe: FAM‑ TTGATGAGTGCACCCAAGATCCA ‑TAMRA |
| human | IL-1β | NM_000576 | Forward primer: CCGACCACCACTACAGCA |
|  |  |  | Reverse primer: AGGGAAAGAAGGTGCTCAGG |
|  |  |  | TaqMan probe: FAM‑ CTGCCCACAGACCTTCCAGGAGAA ‑TAMRA |
| mouse | IL-6 | NM_03168 | Forward primer: CTTCACAAGTCCGGAGAGGA |
|  |  |  | Reverse primer: TCCACGATTTCCCAGAGAAC |
|  |  |  | TaqMan probe: FAM‑ CAGAGGATACCACTCCCAACAGACCTG ‑TAMRA |
| mouse | MCP1 | NM_011333 | Forward primer: CCTGCGGCTTAATTTGACTC |
|  |  |  | Reverse primer: GACAAATCGCTCCACCAACT |
|  |  |  | TaqMan probe:FAM‑ TCTTTCTCGATTCCGTGGGTGGTG ‑TAMRA |
| mouse | αSMA | NM_007392 | Forward primer: CAGGCATTGCTGACAGGAT |
|  |  |  | Reverse primer: GTTCTGGAGGGGCAATGAT |
|  |  |  | TaqMan probe: FAM‑ CTCGCACCCAGCACCATGAAGA ‑TAMRA |
| mouse | CTGF | NM_010217 | Forward primer: TACCGTGGGAGGAACTATCC |
|  |  |  | Reverse primer: CTCACCTCAGTGTGCGTTCT |
|  |  |  | TaqMan probe: FAM‑ CAGTTGTTCATTAGCGCACAGTGCC ‑TAMRA |
| human | NQO1 | NM_000903 | Forward primer: TTCCAGAAAGGACATCACAGG |
|  |  |  | Reverse primer: AGCTTCTTTTGTTCAGCCACA |
|  |  |  | TaqMan probe: FAM‑ TCCTGCCGAGTCTGTTCTGGCTT ‑TAMRA |
| human | HO-1 | NM_002133 | Forward primer: AGGCAGAGGGTGATAGAAGAGG |
|  |  |  | Reverse primer: CAACTCCTCAAAGAGCTGGATG |
|  |  |  | TaqMan probe: FAM‑ CCAAGACTGCGTTCCTGCTCAA ‑TAMRA |
| human | GCLM | NM_002061 | Forward primer: AGTGGGCACAGGTAAAACCA  Reverse primer: AGCAAATGCAGTCAAATCTGG  TaqMan probe: FAM‑ ATCTTGCCTCCTGCTGTGTGATGC ‑TAMRA |
|  |  |  |  |
|  |  |  |  |
|  | 18S rRNA | NR_003278 | Forward primer: CCTGCGGCTTAATTTGACTC |
|  |  |  | Reverse primer: GACAAATCGCTCCACCAACT |
|  |  |  | TaqMan probe: FAM‑ TCTTTCTCGATTCCGTGGGTGGTG ‑TAMRA |
|  | FAM, 6‑carboxyfluorescein; TAMRA, N,N,N',N'‑tetramethyl‑6‑carboxyrhodamine derivative. | | |
